# Increasing incidence of surgically treated hamstring injuries: a nationwide registry study in Sweden between 2001 and 2020

**DOI:** 10.2340/17453674.2023.13650

**Published:** 2023-07-04

**Authors:** Sofia LASZLO, Kenneth B JONSSON

**Affiliations:** Department of Surgical Sciences, Uppsala University, Uppsala, Sweden

## Abstract

**Background and purpose:**

Data on incidence and on trends in treatment of hamstring injuries, including proximal hamstring tendon avulsions (PHA), is limited. We aimed to investigate the incidence, trends in operative treatment, age, and sex distribution of hamstring injuries in Sweden between 2001 and 2020.

**Patients and methods:**

We obtained data recorded in the National Patient Register between 2001 and 2020 on patients between 18 and 90 years of age, with the ICD-10 code S76.3, to calculate the incidence of patients treated operatively for hamstring injuries in Sweden. Patients with the NOMESCO classification NFL49 were considered as having been treated operatively. Data on quadriceps and Achilles tendon injuries were obtained for comparison. To calculate incidences, adult population data for every year were obtained from the Statistics Sweden website.

**Results:**

The incidence of patients diagnosed with hamstring injuries increased from 2.2 to 7.3 per 100,000 person-years. There was a rising trend of surgical treatment per diagnosed case from 3.0% to 14.2%. Patients diagnosed in units with the highest experience of surgical treatment of hamstring injuries tended to be operated on more often (22.2%) than patients diagnosed in units with limited experience (5.1%), although the fraction of operated patients was increasing in both groups.

**Conclusion:**

Between 2001 and 2020 there was an increase in the proportion of operatively treated hamstring injuries.

Injuries to the hamstring muscles of the posterior thigh represent a spectrum of conditions ranging from acute injuries, such as strains to the muscle belly and complete or partial tendon ruptures, to chronic degenerative tears. Although much of the literature on hamstring injuries covers a younger population, injured during athletic activities [1-3], recent data suggests that hamstring injuries are diagnosed frequently in the middle-aged population [4-6].

Hamstring injuries are primarily treated nonoperatively, apart from complete proximal hamstring tendon avulsions (PHAs) that can be treated operatively with reattachment of the tendon to the ischial tuberosity [7,8]. There is still no highquality evidence guiding the treatment choice of PHA [5,9] and the preferred treatment varies between different units [10]. So far, there are no population-based studies on PHA and the incidence of these injuries in the total population is unknown. There is also limited data on the incidence of operative treatment and on trends in the choice of treatment over time.

PHAs share some clinical similarities with other relatively common lower extremity tendon avulsions and ruptures. All are associated with degenerative changes in the musculotendinous complex, suggesting a common pathogenesis [11], and there is often controversy regarding indication for operative treatment [12-14]. Although there are no comparative studies of these common injuries, variability in their epidemiology, e.g., age and sex distribution, may differ.

Our study aims to investigate the age and sex distribution, seasonal variation, and trends in the national incidence of hamstring injuries between 2001 and 2020. We also study possible trends in the choice of treatment modality and whether there are differences in the frequency of operative treatment dependent on the healthcare provider. For comparative and illustrative reasons, we also investigate incidence, proportion operated on, and sex distribution of Achilles and quadriceps tendon injuries.

## Patients and methods

The study was a register-based descriptive study using data from the National Patient Register (NPR). The study is reported according to the RECORD guidelines.

The NPR is a government agency under the Swedish National Board of Health and Welfare and includes information on all inpatient care in Sweden since 1987. The register also covers outpatient doctor’s visits, including day surgery from both private and public caregivers since 2001. Data such as personal identification number, age, sex, patient’s domicile, primary and secondary diagnoses, length of hospital stay, and surgical procedures performed is included in the register. Diagnoses in the NPR have been coded with the International Classification of Diseases, Tenth Revision (ICD-10) since 1997. The register has been validated, and < 1% of the records on somatic care are missing data [15].

The ICD-10 coding system is without a specific code for PHA. In the ICD-10 classification, the code represents the entire muscle or muscle group of the extremity or part of the extremity, i.e., no subclassification is used to identify a specific anatomic location within the same muscle group. There are 6 ICD-10 codes for muscle injuries in thigh and hip. S76.3 (injury of muscle and tendon of the posterior muscle group at thigh) was selected with the aim to investigate hamstring injuries. To verify this selection, we queried the statistical NPR database (www.socialstyrelsen.se) for the distribution of ICD-10 diagnoses among patients treated with suture or reinsertion of tendon in the hip or thigh (NFL49). Only 3% of patients treated with suture or reinsertion of tendon in the hip or thigh (NFL49) had a primary diagnosis not covered by S76.1 (injury of quadriceps muscle and tendon) or S76.3.

**Figure 1:**
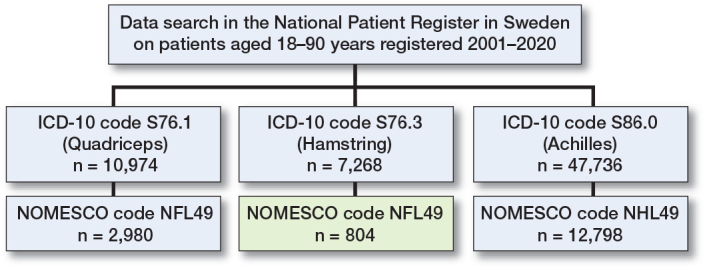
Flowchart of included cases.

We obtained data recorded in the NPR between 2001 and 2020 on all patients between 18 and 90 years with the ICD diagnosis S76.3. Furthermore, we obtained data on 2 other common tendon injuries of the lower extremity using ICD-10 S76.1 (injury of quadriceps muscle and tendon) and S86.0 (injury of Achilles tendon) (Figure 1).

In the output dataset, the procedure coupled to the patient visit was coded with the NOMESCO (Nordic Medico-Statistical Committee) classification and this was used to assess surgical intervention. Patients with ICD-10 code S76.3 in combination with NOMESCO classification NFL49 (suture or reinsertion of tendon of hip or thigh) were considered to represent operatively treated hamstring injury. Patients with ICD-10 code S76.1 in combination with NFL49 were considered to represent operatively treated quadriceps injuries and patients with ICD-10 code S86.0 in combination with NHL49 (suture or reinsertion of tendon of ankle or foot) were considered to represent operatively treated Achilles tendon injuries. In case of multiple visits or hospitalization only the first visit of every patient was counted. It was not possible to determine whether the initial decision was operative treatment or if this was re-evaluated at a later point. To calculate incidences, adult population data for every year was obtained from the Statistics Sweden website. Persons < 18 years and > 90 years were excluded from the analysis. The Statistics Sweden website obtains an electronic register of the population in Sweden for each year [16]. The results are based on the entire adult population of Sweden and are not cohort or sample based.

The main outcome is the incidence of patients treated surgically for hamstring injury with the ICD-10 code S76.3 and NOMESCO-code NFL49 in Sweden between 2001 and 2020.

### Statistics

As this was a purely descriptive national study no statistical comparative calculations were performed. Statistical analysis was performed using R (version 4.0.2; R Foundation for Statistical Computing, Vienna, Austria) software.

### Ethics, registration, data sharing, funding, and disclosures

Ethics approval was obtained from the Ethical Review Board, Stockholm Sweden (Dnr 2020-05499). The study was partially funded by a grant from AFA (Arbetsmarknadens Försäkring-saktiebolag) insurance and by local governmental research funding at Uppsala University Hospital. Data sharing is available upon reasonable request. The authors report no conflict of interest. Completed disclosure forms for this article following the ICMJE template are available on the article page, doi: 10.2340/17453674.2023.13650

## Results

**Figure 2:**
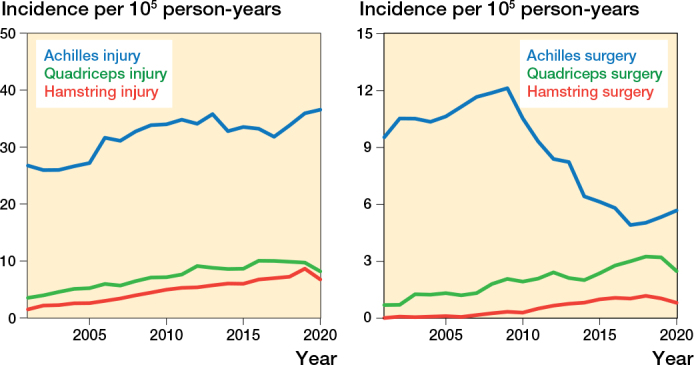
(A, left) Incidence per 105 person-years for Achilles, quadriceps, and hamstring injuries (ICD-10 S86.0, S76.1, and S76.3) and (B, right) incidence of operative treatment of the respective injuries for all Swedish adults aged between 18 and 90 years between 2001 and 2020.

There was an increasing trend in the incidence of all studied injuries (ICD-10 S76.3, S86.0 and S76.1) during the studied time period (Figure 2A).

**Figure 3:**
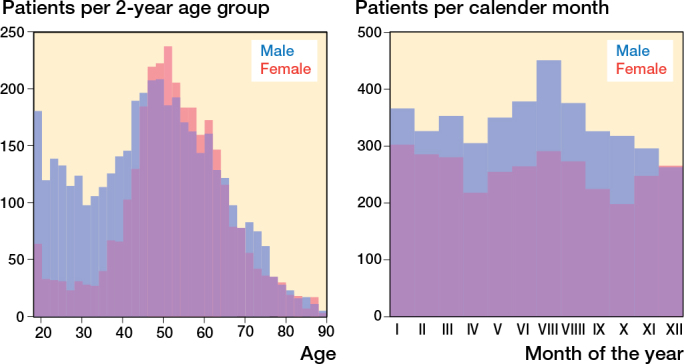
(A, left) Age distribution and (B, right) seasonal variation for time of injury for male and female patients with hamstring injuries (ICD-10 S76.3) in Sweden between 2001 and 2020.

7,268 patients with hamstring injuries aged between 18 and 90 years were identified in Sweden between 2001 and 2020. There were more men (4,137) than women (3,131). The mean age at time of diagnosis was 47.4 years for men and 52.8 years for women (Table). The age and sex distribution of patients with hamstring injury can be seen in Figure 3A. The incidence for different months of the year can be seen in Figure 3B. Compared with the summer months there were 11% fewer injuries in the winter, 13% fewer injuries in the spring, and 21% fewer injuries in the autumn.

**Table:** Number of patients given the ICD-10 diagnosis S76.3, S76.1, and S86.0, the mean age and sex distribution and the number of operatively treated patients (NOMESCO NFL49 and NHL49) in Sweden between 2001 and 2020

ICD-10	Patients	Patients treated operatively	Mean age (SD)	% Male
S76.3 (Hamstring)	7,278	804	49.7 (10.6)	57
S76.1 (Quadriceps)	10,974	2,980	55.1 (12.4)	82
S86.0 (Achilles)	47,736	12,798	50.5 (13.1)	79

**Figure 4:**
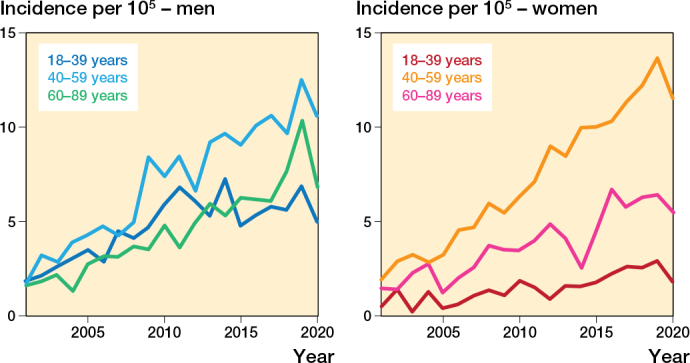
Incidence of hamstring injuries (ICD-10 S76.3) in Swedish adults per 10^5^ person-years between 2001 and 2020 for (A, left) men and (B, right) women by age group.

During the first 5-year period, 2001–2005, 124 men and 64 women had a hamstring injury per year. This increased to 315 men and 265 women per year between 2016 and 2020. The incidence of patients with hamstring injury increased from 2.2 per 10^5^ person-years (p-y) in 2001–2005 to 7.3 per 10^5^ p-y in 2016–2020 (Figure 2A). A similar trend was seen for all age groups, but the greatest increase was in women aged 40–59 years (Figure 4B). In comparison, the incidence of Achilles tendon injuries in 2001–2005 was 26.5 per 10^5^ p-y and in 2016–2020 this was 34.3 per 10^5^ p-y. The incidence of quadriceps tendon injury was 4.5 per 10^5^ p-y in 2001–2005 and 9.6 per 10^5^ p-y in 2016–2020 (Figure 2A).

804 sutures or reinsertions of the hamstring tendon(s) (NFL49) were performed during the study period. There were no patients with the procedural codes NFL39 (myotomy or tenotomy of hip or thigh) or NFL19 (suture or plastic repair of muscle of hip or thigh). The combination of ICD-10 S76.3 and NFL99 (other operation on muscle or tendon of hip or thigh) was found in 17 cases. These were excluded from subsequent analysis.

**Figure 5:**
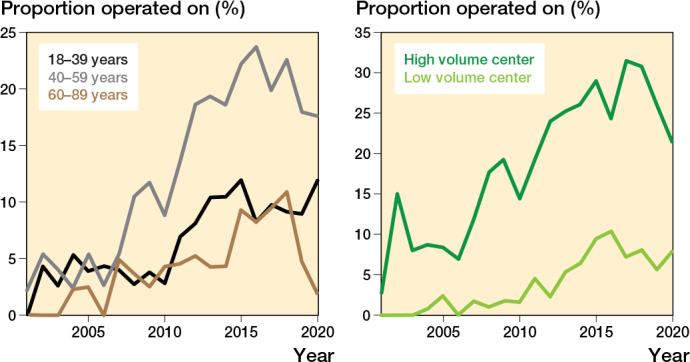
Proportion of patients with (A, left) hamstring injuries (ICD-10 S76.3) operated on in Sweden between 2001 and 2020 by age group, and (B, right) hamstring injuries (ICD-10 S76.3) operated on with the primary contact in high- or low- volume centers between 2001 and 2020.

There was an increase in the incidence of operatively treated hamstring injuries during the studied period (Figure 2B). The total number of operatively treated hamstring injuries per year increased from 5 in 2001–2005 to 82 in 2016–2020. The frequency of surgical treatment per diagnosed case increased from 3.0% in 2001–2005 to 14.2 % in 2016–2020. Patients aged 40–59 had the greatest increase in proportion of surgeries per case as well as incidence (Figures 4 and 5A). In comparison, the percentage of operated Achilles tendon injuries was down from 38.9% in 2001–2005 to 15.6% in 2016–2020 (Figure 2B). For quadriceps tendon injuries the proportion of operated injuries increased from 22.3% in 2001–2005 to 30.7% in 2016–2020.

There was a total of 150 different healthcare providers where patients were registered at time of diagnosis for hamstring injury. Of these, 49 caregivers operated on at least 1 patient. We compared the 10 units with the highest volume in operations with the remaining 140 units. 2,533 patients had their primary contact in the high-volume centers and 4,745 in the low-volume centers. In the high-volume group there were 562 (22.2%) operated patients compared with 242 (5.1%) in the low-volume group. The fraction of operated patients was increasing in both groups during the studied period (Figure 5B).

## Discussion

The main finding of our study is a 3-fold increase in surgical treatment of patients with hamstring injuries between 2001 and 2020. To our knowledge, this is the first study describing a population-based national incidence of operative treatment of hamstring injuries. The study is based on national data covering both inpatient and outpatient care.

The ICD-10 code S76.3 (injury of muscle and tendon of the posterior muscle group at thigh) is unspecific with regards to location and severity of the tendon injury. Surgical treatment of hamstring injuries for reasons other than PHA is rare [17,18], which is why we suggest that the increase in the proportion of surgically treated hamstring injuries reflects an increase in the surgical treatment of PHA.

The frequency of surgical treatment per diagnosed hamstring injury increased from 3.0% in 2001–2005 to 14.2 % in 2016–2020. The rise in the number of surgeries is an effect of both an increase in the incidence of diagnosed injuries and an increase in the proportion of patients treated operatively. The increase in hamstring injuries, as well as the probability of their surgical treatment, is driven primarily by an increase in these injuries among patients aged 40–60 years. A possible explanation for this observation could be that PHA is more prevalent within this group, compared with younger patients who may have other posterior thigh injuries that are treated adequately nonoperatively. Another explanation could be that surgeons have become less reluctant to treat middle-aged patients with PHA operatively.

The increased proportion of operatively treated patients during the study period is noteworthy, as there is no high-quality evidence suggesting that operative treatment of PHA is superior to nonoperative treatment [5,19]. So far, few studies have reported the outcome of nonoperatively treated proximal hamstring injuries and this, together with increasing patient demands and more surgeons with procedural experience, could explain the rise in the number of surgeries [5,19].

We compared trends on hamstring injuries with similar data on the quadriceps and Achilles tendons. These analyses served as an internal control of our data and contributed with previous absent epidemiological comparisons between these tendon injuries. The increase in incidence over time was not limited to hamstring injuries but was seen for all studied tendon injuries. A more unrestrictive approach and better availability of MRI could be a possible explanation, with more injuries being diagnosed [20]. Patients in general could have become more prone to seek healthcare than previously and the awareness of these injuries could have increased in the orthopedic community. A change in referral patterns among general practitioners could also have influenced the propensity to refer patients and thereby cause an apparent increase in incidence. Interestingly too, our study also verified the change in the treatment choice for Achilles tendon injuries over the last decade from operative to nonoperative treatment [21], demonstrating that established treatment traditions may shift in response to the appearance of new high-quality evidence.

Our study demonstrates that tendon/muscle injuries are overrepresented in the male population. Achilles and quadriceps injuries were diagnosed in males 4 out of 5 times. Compared with the other studied tendon injuries, hamstring injuries are more equally distributed among men and women (43% women). In the middle-aged patients there was a higher proportion of women with hamstring injuries. The reason behind this finding is not apparent. In comparison with Achilles and biceps brachii tendon injuries, the mechanism of injury could be different, with more of an uncontrolled lengthening of the muscle rather than failure during contraction. Therefore, muscle mass could perhaps be a risk factor for Achilles injuries but not for hamstring injuries. On the other hand, women are known to have higher hamstring muscle extensibility in general [22], which should protect against injury during uncontrolled lengthening. More research will be necessary to refine and further elaborate these findings.

Our study shows that more surgeries are performed for hamstring injuries, despite lack of evidence. Our findings suggest inequalities within the national healthcare system, such that the likelihood of operative treatment is dependent on the caregiver where the injury is primarily diagnosed (Figure 4). Recently, better comparative studies have been published and a large randomized controlled study is under way [5,19,23]. Based on this novel data, future guidelines on the treatment of hamstring injuries will have to address the inequalities and potentially harmful trends identified in this study.

### Limitations

The greatest limitation in this study is that the ICD coding does not allow any further specification of the anatomical site or specification of the extent for the hamstring injury. ICD-10 S76.3 therefore covers all forms of hamstring injuries, and it is not possible to directly determine the incidence of PHA.

Several types of misclassifications are also possible. Diagnostic errors, translation errors, and coding errors are present in the NPR (15). Positive predictive values of the inpatient register (part of NPR) have been found to be 85–95% for many diagnoses but are not available for current injuries. Other uncontrollable confounding risks include changes in coding praxis over time or differences in coding praxis among different healthcare providers. Nonetheless, we believe to have limited the risks of confounders influencing our outcomes especially in the estimate of operative treatment of tendon injuries, as it is known that the surgical procedural codes are reliable in the registry [24].

In the study, we have, of necessity, chosen diagnostic and procedural codes and defined the assumed injury and treatment represented by these. For all injuries we present treatment data based on the NOMESCO NxL49 (suture or reinsertion of tendon) codes. We chose this narrow definition, as opposed to including all procedural codes, as we aimed at describing incidences of tendon repairs. It is plausible that other type of injuries, such as intramuscular hematomas, would be coded by the more unspecific codes.

Finally, our interpretation that operatively treated patients (NFL49) with the S76.3 diagnosis represent operatively treated PHA is based on the authors’ assumption that surgical treatments of other injuries in the back of the thigh are very rare. A small number of cases with ICD-10 code S76.3 and NOMESCO NFL49 that do not fall into the category of PHA are probably included.

### Conclusions

Hamstring injuries were equally distributed among men and women, but there was a higher proportion of women with hamstring injuries among the middle-aged patients. There were more patients with hamstring injuries during the summer, compared with the rest of the year. There was an increasing trend in the incidence of hamstring injuries between 2001 and 2020. There was also an increase in the proportion of operatively treated hamstring injuries. This is likely due to an increase in operative treatment of PHA, despite lack of evidence supporting this treatment.

## Notes

SL and KBJ were responsible for the study design and retrieving of data from Socialstyrelsen. Analysis of all data was done by SL, as well as drafting the manuscript. KBJ and SL edited and approved the final version of the manuscript.

Handling co-editors: Jeppe Vejlgaard Rasmussen and Robin Christensen

*Acta* thanks Gino Kerkhoffs and Jeppe Lange for help with peer review of this manuscript.
